# The Impact of Technology on Safe Medicines Use and Pharmacy Practice in the US

**DOI:** 10.3389/fphar.2018.01361

**Published:** 2018-11-20

**Authors:** Philip J. Schneider

**Affiliations:** ^1^MediHealthInsight, Scottsdale, AZ, United States; ^2^Division of Pharmacy Practice and Science, College of Pharmacy, The Ohio State University, Columbus, OH, United States

**Keywords:** hospital pharmacy, medication therapy management, clinical pharmacy, medication technology, patient safety

## Abstract

For decades it has been suggested that pharmacists are under-utilized and could better use their knowledge and experience to improve the use of medicines. The traditional roles for pharmacists have been preparing and distributing medicines, but this has limited both the location where they work and the available time to work more closely with other healthcare professionals to improve both the effectiveness and safety of medicines. Newly emerging technologies have made this possible. Examples include robotics that automate preparation and distribution of medicines, electronic health information, clinical decision support systems, and machine readable coding on medicine packaged. As a result of the use of these technologies, pharmacists in hospitals are working outside the hospital pharmacy and spending more time in medication therapy management activities compared to traditional distribution roles.

## Introduction

The adoption of innovative ideas can be painfully slow, even when an innovation has well-demonstrated positive impact. In his work Diffusion of Innovation, Rogers concluded that based on a study of the adoption of new ideas, it takes decades for an innovation to be widely accepted (Rogers, 2003). One would like to think that when the public clearly benefits from a technology that the adoption rate would be quicker. In health care, where innovations have the potential to improve the effectiveness, safety, and efficiency of care, the imperative for change would seem to be clear. As we will see, this is not always the case.

## What We Know About Medicines Use

The American Society of Health-System Pharmacy (ASHP) is an organization representing pharmacists practicing in hospitals and organized health care settings. For more than 50 years, ASHP has surveyed hospital pharmacy directors in US hospitals beginning with the landmark publication Mirror to Hospital Pharmacy in 1965 (Francke et al., 1964). This report assessed the practice of pharmacy in US hospitals. This audit revealed a scope of service that was limited to drug preparation and distribution. The authors challenged the profession to aspire to a more professional role by strengthening the relationship between physicians and pharmacists to improve the use of medicines.

Since then, more than 20 such surveys have been conduced. Since 1998, this survey has been conduced annually and the results provide the opportunity to identify, track, and trend changes in medicines use practices and the role of pharmacists in hospitals (Ringold et al., 1999, 2000; Pedersen et al., 2001, 2003, 2004, 2005, 2006, 2007, 2008, 2009, 2010, 2011, 2012, 2013, 2014, 2015, 2016, 2017; Schneider et al., 2018). Through these surveys, progress toward the vision described in Mirror to Hospital Pharmacy has been largely realized, albeit in a time frame that supports Rogers’ theory of the diffusion of innovations. Much of this progress has been through the use of technology.

At the inception, these surveys were intended to determine what pharmacists were doing in hospitals and to assess the scope of pharmacy services in the US. In 1998, the surveys were reformatted to focus on the steps in the system of medicines-use, not just pharmacy practice. It acknowledged that this system includes many participants, not just pharmacists. At the time, a widely published report from the Institute of Medicine titled To Err is Human called attention to harm resulting from care intended to help patients (Institute of Medicine, 2000). This report focused on problems with the system of care as being more significant than the performance of individual health care professionals. That was the rationale for changing the format of the survey.

## The Epidemiology of Problems With Medicines Use

The medicines use system is at least multidisciplinary but ideally an interdisciplinary system. While the roles of health care professionals overlap and can vary, typically a physician prescribes a medicine, a pharmacist prepares and dispenses the dose, and the nurse, patient or family member administers the drug. The performance of each member of this team can affect the effectiveness, safety, and efficiency of a medicine and its use, but so can the interaction of these participants in the system, each of whom functions at different places and times in the process. Problems with handoffs and communication often contribute to errors, adverse events and a loss of efficiency. It would seem obvious that technology could improve the performance of the system if medicines use to the benefit of patients, health care professionals, and the institution in which the patients are cared.

Problems with the performance of the medicines use system have been studied. A seminal study that called attention to problems with the use of medicines was the Harvard Medical Practice study. The results of a review of more than 30,000 patient records found that 3.7% of hospitalized patients were injured from the care that they received (Brennan et al., 1991). The most common injury was “drug complications,” which accounted for 19% of all events detected (Leape et al., 1991)In a follow up study of medication errors in hospitalized patients, it was found that mistakes resulting in harm to patient occurred by all participants in the medicines use system and at all steps in the system. The most common step where errors occurred was when medicines were prescribed. Fully19% of the mistakes that resulted in harm to patients were detected at this step. The next most common step was when a nurse administered a medicine to a patient, where 17% of the errors occurred. Less common steps where errors occurred was during the transcribing of orders for medicines and when doses were prepared (Bates et al., 1995). These investigators looked further to determine what “system failures” contributed to the medication errors. The two most common underlying causes were a lack of information about the patient (e.g., unknown allergy to a drug) and a lack of information about the drug (e.g., an need to adjust the dose in certain patients) (Lucian et al., 1995). It is easy to see how technology can be used to address these system problems.

## Safe Practices for Medicines Use

Well-before the Harvard Medical Practice study, problems with drug administration errors in by nurses to hospitalized patients were documented. Studies showed error rates of up to 10% occurred when comparing what was prescribed to what was actually administered to the patient (Barker, 1969). The “system” of medicines use in place at the time this study was conducted was one where all drugs were stocked in bulk containers on nursing units in the hospital. A “medication nurse” would prepare a medication tray by taking individual doses from the bulk supply, placing them in individual containers for each patient on the unit, and going from room to room to administer the doses at the scheduled time. With this method, the nurse performed both a dispensing and drug administration role and was expected to administer what the physician ordered with out questioning it. A new system that included more double checks and safeguards called the “unit dose” system was shown to reduce medication errors by as much as 50%. This system transferred responsibility for dispending medicines from the nurse to the pharmacist, providing an additional double check by the pharmacist in the process. It also provided a limited supply (24 h or less) of individually packaged and labeled medicines in a patient-specific container for the nurse to use when administering medicines (Barker, 1969). This made it less likely that the wrong dose or wrong drug would be administered or the medicine administered to the wrong patient or at the wrong time.

**FIGURE 1:**
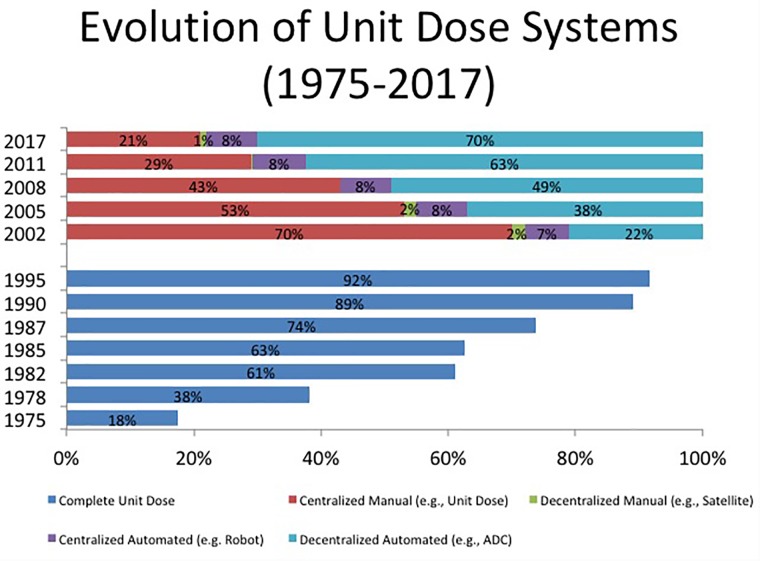
Trends in drug dispensing.

**FIGURE 2:**
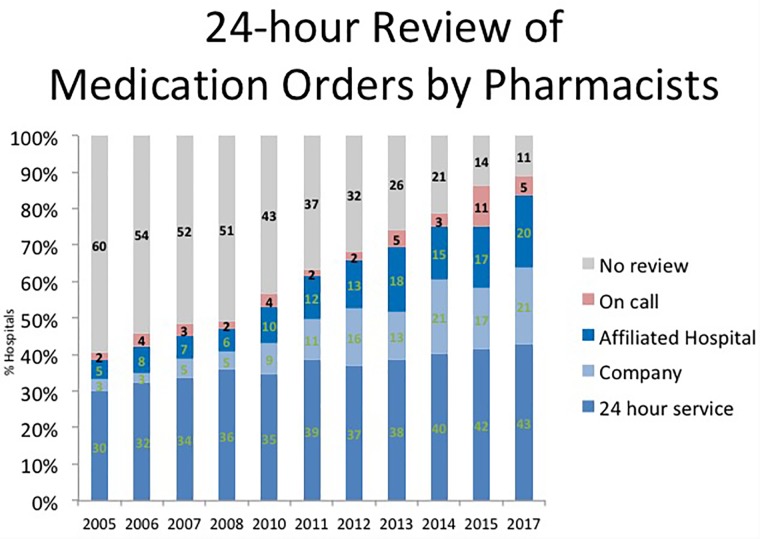
Twenty Four-hour review of medication orders by pharmacists.

While evidence supporting the safety of unit dose drug distribution system was published in 1968, the adoption of this innovation followed the timeline suggested by Rogers in Diffusion of Innovation. For example, in 1975, unit dose drug distribution systems were in place in only 18% of US hospitals. It took until 1995; 20 years for this system to be adopted in 92% of hospitals – consistent with the timeline of Rogers (Schneider et al., 2018) (Figures 1, 2).

## Technology Enabled Changes

There are opportunities to harness new technologies to improve the use of medicines and transform pharmacy practice. Baines and colleagues has presented a conceptual framework for analyzing production methods, productivity and technology in pharmacy practice that differentiates between dispensing and pharmaceutical care services. They outline a framework to study the relationship between pharmacy practice and productivity, shaped by educational and technological inputs (Baines D. et al., 2018).

While the transfer of responsibility for dispensing of medicines from nursing to pharmacy had a positive impact on patient safety, it did not improve operational efficiency. Preparing patient-specific medication bins for each patient every day (if not many times per day) was much more labor intensive and expensive. It also created a lag between when medications were prescribed and when they were available to the nurse for administration to the patient. Medication orders change during the day, making the unit dose bins from the pharmacy outdated. This became worse as acuity increased and length of stay decreased. Two technology enabled changes resulted: robotic filling of unit dose bins and stocking medications in patient care areas using automated dispensing cabinets.

While robotic enabled centralized unit dose cart filling systems solved the workforce issue and improved accuracy in dispensing, it did not solve the responsiveness to order changes. Moreover, robotic systems were very expensive and their use limited to very large hospitals. What became a more popular option was re-locating medications to the patient care areas using automated dispensing cabinets. To illustrate this, only 7% of US hospitals employed robotic technology in 2002 for centralized unit dose drug distribution systems. This remained steady over time and only 8% had this technology by 2017 (Schneider et al., 2018). In contrast, 22% of US hospitals used automated dispensing cabinets in patient care areas for drug dispensing in 2002, but this rose to 70% by 2017. A summary of these trends is shown in Figure 1. Robotic technology could also used to compounding sterile preparations in the pharmacy. This technology is quite expensive and does not enjoy widespread use in US hospitals. The percentage using this technology has remained steady at less than 3% in the 6 years that this has been surveyed (Schneider et al., 2018). Robotic technology is more commonly used to compound more complex nutrition support formulations. These preparations are not only time consuming to compound, but errors are more likely to result in harm to patients. In 2017, 14.3% of hospitals used robotic technology to compound nutrition support formulations (Schneider et al., 2018).

Reverting back to a floor stock system, albeit a technology enabled one created the potential to risk an increase in medication errors comparable to the rate documented with the traditional floor stock system. Again – technology to the rescue. Two innovations emerged to prevent a return to unacceptable medication error rates. The first was the “profiling system” where a pharmacist review and approval of the prescribed therapy was necessary before a nurse could access a medicine from an automate dispensing cabinet. The second was “lidded pockets” where access to any container within the automated dispensing cabinet is restricted so that a nurse does not have free access to all medicines in the automated dispensing cabinet; only the pocket that has the medicine ordered for that patient. The use of lidded pockets has increased from 51.8% in 2008 to 70.1% in 2017 (Schneider et al., 2018).

A review of prescription orders by a pharmacist is considered a safe medication practice. Lesar, at all found 3 errors per 1000 prescriptions detected by hospital pharmacists (Lesar et al., 1997). A double check by pharmacist is important to detect and prevent errors in prescribing causing an adverse drug event. Unit dose and decentralized automated dispensing cabinet- based systems with profiling offer this double check before a dose is made available for administration to the patient. Historically, this practice required a pharmacist to be physically present in the hospital to review prescriptions and a 24-h pharmacy service, which was expensive and not always possible in smaller hospital. The advent of technologies including the electronic health record and properly configured automated dispensing cabinets has made it possible for a pharmacist to review and approve prescriptions remotely before a nurse can obtain and administer a medicine to a patient. As a result, the percentage of hospitals where a pharmacist does not review prescriptions before a medicine is available for administration to a patient has continued to decline from 60% in 2005 to 11% in 2017 as shown in Figure 2 (Schneider et al., 2018).

Computer prescriber order entry systems were thought to reduce the need for pharmacist to review medication orders before doses were available for administration to the patient. These systems employ clinical decision support, which alert prescribers to potential dosing errors, drug allergies and drug interactions. Early investigators considered this a “systems solution” to address some of the more common underlying causes of prescribing errors; namely lack of information about the patient and the drug prescribed (Bates et al., 1995; Lucian et al., 1995). Between 2003 and 2016, the percentage of US hospitals with computer prescriber order entry systems with clinical decision support increased from 2.5 to 95.6% (Pedersen et al., 2017). While electronic prescribing has become almost universal, problems with alert fatigue and the low positive predictive value of alerts has limited the impact of these systems on error rates and has not eliminated the value of a pharmacist review of medication orders.

Another technology that has been show to improve safety and efficiency is machine-readable bar coding of medication packages. This technology has been used in many industries to more accurately reconcile and verify the identity of objects and would have logical application in verifying the identity of medicines, the persons handling them, and the patient to whom they are administered. This technology has been used in both the pharmacy to improve the safety and efficiency of drug storage, preparation, and dispensing, and by nursing to improve the safety and efficiency of drug administration and documentation in the medication administration record. Barcode scanning is also used to verify ingredients when sterile preparations are compounded in the pharmacy. The percentage of hospitals that are doing this has increased from 11.9% in 2011 to 26.9% in 2017 (Schneider et al., 2018). The use of machine-readable coding to verify the accuracy of drug dispensing has increased from 5.7% in 2002 to 61.9% in 2017 (Schneider et al., 2018). This technology is also used to verify the accuracy of restocking automated dispensing cabinets in patient care areas. The percentage of hospitals doing this has increased from 43.3% in 2011 to 74.7% in 2017 (Schneider et al., 2018). Bedside bar code reconciliation of doses during drug administration by nurses enjoys widespread use. In 2016, 92.6% of US hospitals used this technology; an increase from only 1.5% in 2002 (Pedersen et al., 2017).

## How Has This Transformed Pharmacy Practice in Hospitals?

Dating back to Mirror of Hospital Pharmacy, there has been a commitment to advancing the role of pharmacists to improve the use of medicines in hospitals (Rogers, 2003). To that end, ASHP and the ASHP Research and Education Foundation sponsor the Practice Advancement Initiative (PAI) (American Society of Health-System Pharmacists, 2018). The goal of this initiative is to significantly advance the health and well-being of patients by supporting futuristic practice models that support the most effective use of pharmacists as direct patient care providers (Baines D.L. et al., 2018).

A newer role for pharmacists is medication therapy management either by standing protocol or prescriber order/delegation or pharmacists have responsibility for writing medication orders, selecting doses, ordering appropriate laboratory tests, and monitoring patient response to therapy. In 2016, pharmacists managed the following therapies: vancomycin (94% of hospitals), renal dosing of antibiotics (83.9%), aminoglycosides (83.8%), anticoagulants (71.1%), nutrition support (46.9%), selection of antibiotics (19.6%), and pain management (6.2%). These percentages were higher than they were in 2013 (Pedersen et al., 2017). The impact of pharmacist medication management services is measured by the following indicators: cost saving (61.5% of hospitals), patient outcomes (36.5%), federal quality of care indicators (23.7%), readmission rates (16.6%), and patient satisfaction scores (15.8%), decreases in length of stay (8.3%) (Pedersen et al., 2017).

The transition of the pharmacist role from drug preparation and distribution to medication therapy management has resulted in their practice moving from a central pharmacy to the patient care areas. The following clinical areas commonly have pharmacists routinely assigned to manage therapies to a majority of patients at least 8 h/day, 5 days/week: inpatient medical-surgical (43.5% of hospitals). critical care (43.5%), oncology (37.5%), cardiology (32.9%), pediatrics (24.1%), and the emergency department (21.0%) (Pedersen et al., 2016).

Besides enabling a transition in drug preparation and dispensing, technology also enables medication therapy management by pharmacists. Not all patients can or need medication therapy monitoring by pharmacists. A total of 43.4% of hospitals use computerized data mining to identify patients in need of monitoring. Some electronic health records have data mining functionality (58.6%), and others use proprietary clinical surveillance software (28.4%) to compile data needed to identify patients for daily monitoring by pharmacists (Pedersen et al., 2016).

The transition of the patient from the hospital to the community (and back) is a step in health care where handoffs are missed and miscommunication occurs. Pharmacists are also becoming increasingly involved in transitions of care programs to reduce errors and improve care. Some examples of transitions of care activities by pharmacists include: use of medication histories at admission (74.9% of hospitals; in 2016 up from 54.3% in 2002), discharge medication counseling by pharmacists (46.4% from 21.7%), participation in discharge planning (35.8% up from 23.7%), handoff to community pharmacy at discharge (18.3% up from 917%), and designing a patient-specific medication-related action plan (11.2% up from 5.3%) (Pedersen et al., 2017).

As a result of this change in the role of pharmacists, the percentage of them that they spend in drug distribution is now less than 20%. They spend more than 40% of their time reviewing and verifying prescription orders and almost 25% of their time on other clinical activities, including medication therapy management. Besides changes enabled by technology, pharmacy technicians are widely used in hospital pharmacy departments to support that role and activities of pharmacists. Pharmacy technicians spend almost 80% of their time in traditional drug preparation and distribution (Pedersen et al., 2016).

Transitioning from a traditional drug preparation and dispensing role to a clinical role in medication therapy management has had implications for developing the hospital pharmacy workforce. Beginning in 2000, all US pharmacy graduates receive the Doctor of Pharmacy (PharmD) degree that prepares them for the increasing clinical roles that all pharmacists are realizing. These clinical roles are more common and often more advanced in the hospital setting, and additional training is available and increasingly required. These include post-graduate pharmacy residency training; both pharmacy practice (PGY1) and specialty (PGY2) programs. Board certification is also available to assess the competency of pharmacists in selected specialty practice areas through the Board of Pharmaceutical Specialties. Board certification is also available for pharmacy technicians through the Pharmacy Technician Certification Board. At present, 29.4% of hospital pharmacists have completed a PGY1 residency, 8.1% a PGY2 residency, and 23.1% are board certified. For pharmacy technicians the percentage that are board certified is much higher (77.8%) because there is no standard degree awarding program to prepare pharmacy technicians (Schneider et al., 2018).

## Summary

There is currently a need for high quality evaluation of new technologies undertaken in a pharmacy-related setting. We aim to evaluate the use of these monitoring technologies performed in this setting. Worldwide, few evaluations of mobile health, telehealth, smart pump, and monitoring technologies in pharmacy-related setting have been published. Their quality is often below the standard necessary for inclusion in a systematic review mainly due to inadequate study design. Despite the improvements in technology, there is limited evidence on how this translates to real settings and to consumer satisfaction. Most technology driven systems required significant funding and support, particularly those involving latest technology. Rigorous comparative studies are needed to evaluate the effectiveness of different technologies (Baines D.L. et al., 2018).

Nevertheless, voices within the profession of pharmacy have long called for a more important role for the pharmacist. More recently, the public began to call for improvements in the quality of health care, particularly patient safety. New systems of care, many enabled by new technologies have the potential to improve the effectiveness, safety and efficiency of health care, and transform the roles of health care professionals including pharmacists. Unfortunately, the adoption of change is slow, and even though the health of the public is at stake, change in health care is no exception. Over the past decades, however new technologies have enabled the pharmacist to devote more time to working with other health care professionals to improve the use of medicines. Since virtually every patient in the health care system receives medicines, and there is ample evidence that the use of medicines needs to improve, this is a good thing.

## Author Contributions

The author confirms being the sole contributor of this work and has approved it for publication.

## Conflict of Interest Statement

PS was employed by the company MediHealthInsight. The author declares no competing interests.
